# Hodgkin Lymphoma: Biology and Differential Diagnostic Problem

**DOI:** 10.3390/diagnostics12061507

**Published:** 2022-06-20

**Authors:** Taishi Takahara, Akira Satou, Toyonori Tsuzuki, Shigeo Nakamura

**Affiliations:** 1 Department of Surgical Pathology, Aichi Medical University, Nagakute 480-1195, Japan; satoakira@aichi-med-u.ac.jp (A.S.); tsuzuki@aichi-med-u.ac.jp (T.T.); 2 Department of Pathology and Laboratory Medicine, Nagoya University, Nagoya 464-8601, Japan; snakamur@med.nagoya-u.ac.jp

**Keywords:** Hodgkin lymphoma, genetics, tumor microenvironment

## Abstract

Hodgkin lymphomas (HLs) are lymphoid neoplasms that are morphologically defined as being composed of dysplastic cells, namely, Hodgkin and Reed–Sternberg cells, in a reactive inflammatory background. The biological nature of HLs has long been unclear; however, our understanding of HL-related genetics and tumor microenvironment interactions is rapidly expanding. For example, cell surface overexpression of programmed cell death 1 ligand 1 (CD274/PD-L1) is now considered a defining feature of an HL subset, and targeting such immune checkpoint molecules is a promising therapeutic option. Still, HLs comprise multiple disease subtypes, and some HL features may overlap with its morphological mimics, posing challenging diagnostic and therapeutic problems. In this review, we summarize the recent advances in understanding the biology of HLs, and discuss approaches to differentiating HL and its mimics.

## 1. Introduction

Hodgkin lymphoma (HL), initially called “Hodgkin’s disease”, was first reported in 1832 [1]. HLs primarily affect lymph nodes, and are characterized by a mixture of large dysplastic tumor cells and small non-neoplastic inflammatory cells. The biological nature of HLs has long been a mystery, as the neoplastic cells lack both B-cell markers and T-cell markers in most cases. Based on their constellations of morphologic and biologic properties, nodular lymphocyte-predominant HL (NLPHL) and classic HL (CHL) are currently recognized as distinct disease entities, although they share a paucity of neoplastic cells and a rich inflammatory background of non-neoplastic cells, mainly T cells. NLPHL expresses B-cell markers and retains a B-cell phenotype [2], which led to its recognition as a B-cell neoplasm overlapping with T-cell/histiocyte-rich large B-cell lymphoma (THRLBCL). In the International Consensus Classification of Mature Lymphoid Neoplasms (ICC classification), NLPHL was renamed as nodular lymphocyte-predominant B-cell lymphoma [3]. CHLs exhibit reduced expression of B-cell markers, although they are reportedly derived from crippled germinal center (GC) B cells. Infection with Epstein–Barr virus (EBV) plays a significant role in the lymphomagenesis of a subset of CHL. Accumulating evidence indicates that this disease phenotype is characterized by the interaction of immune cells in the microenvironment with neoplastic cells expressing immune checkpoint proteins, represented by CD274/PD-L1. In the present review, we summarize recent advances that have improved our understanding of HL pathogenesis, and describe several morphological mimics of HLs that can be diagnostic pitfalls.

## 2. Classical Hodgkin Lymphoma

Hodgkin lymphomas (HLs) are historically defined based on morphological characteristics. Around 90% of all HLs are CHL [4], which is characterized by tumor cells—namely, mononuclear Hodgkin cells and multinucleated Reed–Stenberg (HRS) cells—with the background of a variable mixture of reactive immune cells, including small lymphocytes, eosinophils, neutrophils, histiocytes, and plasma cells. The HRS cells of CHL usually show B-cell antigen loss, even though GC B cells are considered to be the cellular origin of CHL in most cases. In the revised 4th edition of WHO classification and also in the ICC classification, CHL is subdivided into four histological subtypes—nodular sclerosis CHL (NSCHL), lymphocyte-rich CHL (LRCHL), mixed cellularity CHL (MCCHL), and lymphocyte-depleted CHL (LDCHL)—each of which has a distinct epidemiology, biology, and prognosis.

### 2.1. Epidemiology

The overall average age-adjusted incidence of CHL is 2–3 per 100,000 individuals in western populations, and lower in Asian populations [5,6]. NSCHL, the most common subtype of CHL, shows its peak incidence in adolescents and young adults (AYAs) [7]. MCCHL, the second-most common subtype in Western populations, has its peak incidence rates in the pediatric age group and among elderly adults [8]. The risk of CHL development is associated with socioeconomic status, especially among AYAs [4,9]. High socioeconomic status and lack of exposure to microorganisms during childhood have been suggested to increase the risk of NSCHL development [10,11]. Conversely, MCCHL in the AYA age group—which corresponds with a high prevalence of EBV infection—is predominant in developing countries, and its morbidity decreases with economic development [12]. Regardless of histological subtype, CHL shows male predominance [4,7]. Excluding male predominance, no etiological factors have been identified as associated with LRCHL and LDCHL, due to their rarity [7].

### 2.2. Clinical Features

Most patients with CHL present with asymptomatic lymphadenopathy or a mass on chest radiograph [13]. The most frequently involved site is a cervical lymph node (75% of cases), followed by the mediastinal, axillary, and para-aortic regions. The disease progression typically follows the physiological direction of lymphatic flow [14]. Involvement of non-axial lymph nodes, such as mesenteric and epitrochlear lymph nodes, is rare. Mediastinal involvement is a typical presentation of NSCHL, detected in 80% of these patients. Primary extranodal involvement may be found in immunocompromised hosts. Bone marrow involvement is more frequent in LDCHL than in other subtypes [15]. MCCHL commonly exhibits abdominal and/or splenic involvement. Among the four subtypes, LDCHL shows the poorest prognosis, followed in order by MCCHL, NSCHL, and LRCHL [16].

### 2.3. Histological Feature

RS cells are the diagnostic hallmarks of CHL. Classical RS cells feature large cell morphology (up to 100 μm) with abundant cytoplasm, and are binucleated or bilobed, often appearing as a “mirror image”. Each nucleus contains huge viral inclusion-like eosinophilic nucleoli, sometimes having an “owl’s eye” appearance. The mononuclear variants are called Hodgkin cells. HRS cells frequently exhibit degenerative morphology with condensed nuclear chromatin, and are referred to as mummified cells. However, these cells are not specific to CHL, and can also be observed in other EBV-associated B-cell lymphoproliferative disorders [17]. 

#### 2.3.1. NSCHL

**Figure 1:**
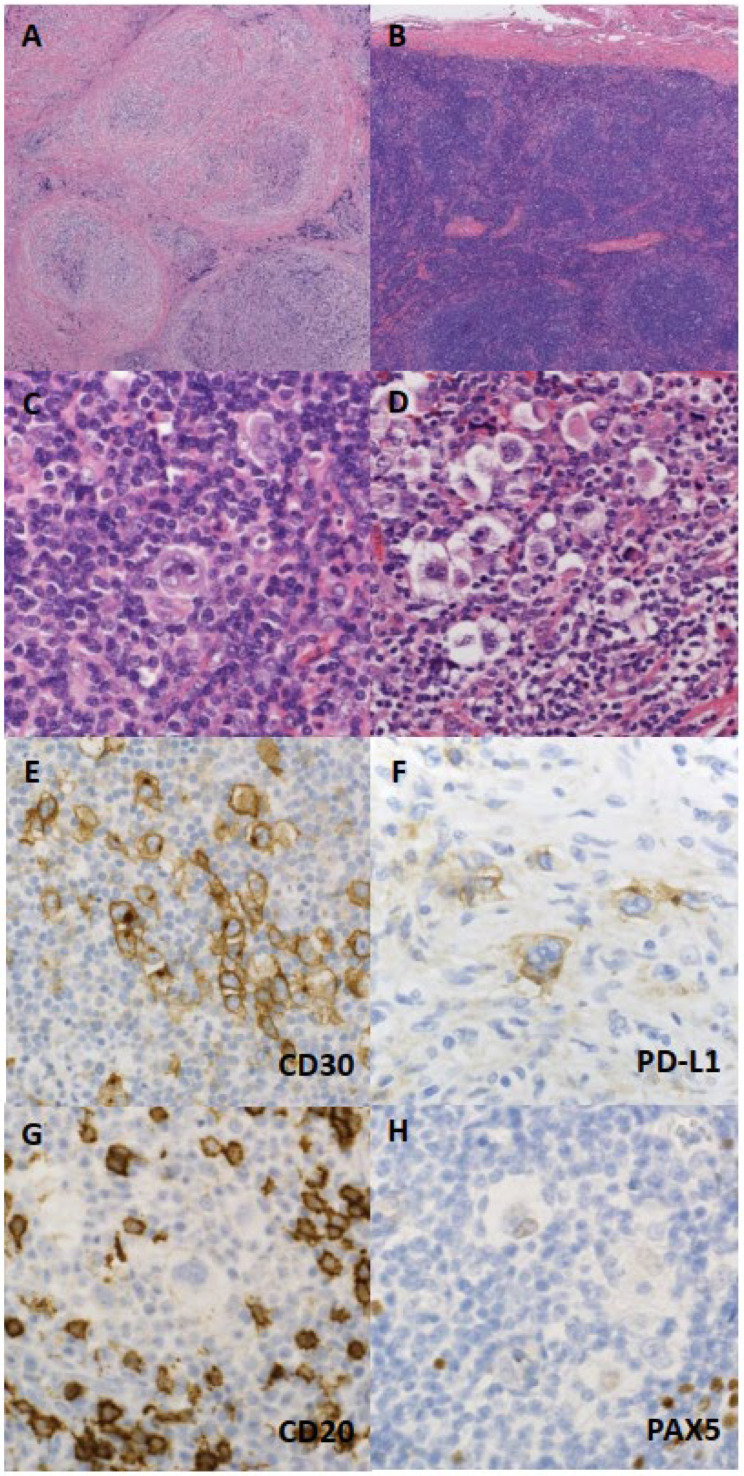
Histological and immunohistochemical features of nodular sclerosis classical Hodgkin lymphoma (NSCHL). (**A**) Cellular nodules are separated by collagen bundles. (**B**) Sclerotic thickening of the lymph node capsule is shown. (**C**) Neoplastic cells that are binucleated with a “mirror-image” appearance, called “Reed–Stenberg cells”. (**D**) Neoplastic cells with a lacuna-like space around the cytoplasm, called “lacunar cells”. (**E**–**H**) Neoplastic cells expressing CD30 and PD-L1 (assessed using clone SP142), lacking CD20 expression, and with very weak PAX5 expression.

NSCHL is characterized by a nodular growth pattern, with each nodule surrounded by collagen bands. Although the presence of fibrosis is a defining feature of NSCHL, the degree of fibrosis is extremely variable—ranging from sclerotic thickening of the lymph node capsule without collagen bands, to total lymph node involvement by sclerotic bands showing almost complete obliterative fibrosis [18] (Figure 1A,B). In the revised 4th edition of the WHO classification system, at least one nodule completely surrounded by collagen bands is required for the diagnosis. The typical tumor cell of NSCHL is called a “lacunar cell”. Lacunar cells have abundant clear to slightly eosinophilic cytoplasm, which is condensed in the perinuclear region, with a lacuna-like space formed around the cytoplasm [17] (Figure 1C,D). Paradoxically, their nucleoli are often indistinct. NSCHL exhibits a background of highly inflammatory cells, including small lymphocytes, and other non-neoplastic cells.

In rare cases, termed “syncytial variant”, neoplastic cells with increased cellular pleomorphism form aggregates, accompanied by a prominent inflammatory reaction and areas of necrosis [19]. In other cases, termed “fibrohistiocytic variant”, fibroblasts and histiocytes with massive sclerosis are abundant, and neoplastic cells may be difficult to identify. These variants are suggested to be associated with poorer prognosis [20]. NSCHL can be histologically graded into two categories, NS1 and NS2, based on the presence of lymphocyte depletion and the number of HRS cells, as historically proposed by the British National Lymphoma Investigation (BNLI) [21,22]. The syncytial and fibrohistiocytic variants are categorized as NS2. However, this BNL grading system is not mandatory in the current WHO classification due to past reports that it lacks consistency. 

#### 2.3.2. MCCHL

**Figure 2:**
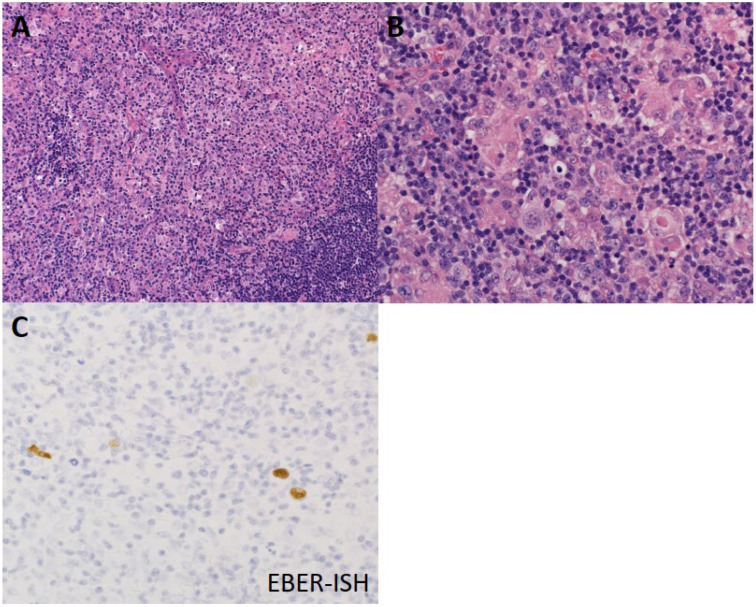
Histological features of mixed cellularity classical Hodgkin lymphoma (MCCHL). (**A**) Granuloma formation by histiocyte aggregation is observed in the background. (**B**) Neoplastic cells are scattered against a background rich in histiocytes and lymphocytes. (**C**) Neoplastic cells with highlighted Epstein–Barr virus RNA through in situ hybridization (EBER-ISH).

In MCCHL, lymph node architecture is usually obliterated—being affected by a mixed population of HRS cells, lymphocytes, plasma cells, eosinophils, and histiocytes (Figure 2A,B). Although interstitial fibrosis may be present, the lymph node capsule is usually not thickened and exhibits no broad band of fibrosis [4]. MCCHL frequently includes EBV-positive tumor cells, which may be accompanied by epithelioid granulomas [23] (Figure 2C).

#### 2.3.3. LDCHL

LDCHL features relatively predominant HRS cells, and scarce background lymphocytes [24]. LDCHL and MCCHL share histological features [25], with both having a high prevalence of EBV-harboring HRS cells, possibly associated with aggressive clinical behavior, and appearing to represent a biologically continuous spectrum [15]. Previously, in Lukes–Butler classification, LDCHL was divided into two histological patterns: diffuse fibrosis and reticular type [26]. The former pattern is characterized by dense fibrosis with prominent fibroblastic proliferation, and the latter by numerous large pleomorphic HRS cells observed with a background of small lymphocytes, histiocytes, and occasional plasma cells [27]. 

#### 2.3.4. LRCHL

LRCHL morphologically mimics NLPHL, making it hard to make this differential diagnosis based on histopathology alone. In contrast with NLPHL, the neoplastic cells of LRCHL exhibit a classical HRS immunophenotype and do not express B-cell antigens, such as CD20 [28]. Similar to NLPHL, most LRCHL exhibit nodular growth containing small reactive lymphocytes and scattered HRS cells. These nodules usually lack neutrophils, eosinophils, and GC B cells corresponding to centrocytes and centroblasts. Rarely, the neoplastic cells show diffuse growth or growth within the interfollicular area [29]. Patients with the diffuse growth variant have a high incidence of relapse, and the distinction from THRLBCL may be problematic [29]. 

### 2.4. Immunophenotype

In almost all CHL cases, the HRS cells express CD30, which is a member of the TNF–nerve growth factor (NGF) receptor superfamily of cytokine receptors, and has been used as a conventional diagnostic marker [30] (Figure 1E). However, many other lymphoid neoplasms also show CD30 expression [31,32,33]. Another diagnostic marker is CD15, which is expressed on HRS cells in the majority of CHL cases (75–85%). Notably, the lack of CD15 expression may be associated with poorer prognosis [4,34]. PD-L1 is a novel diagnostic marker of CHL, which is expressed in 73–96% of CHL cases [35,36,37,38,39] (Figure 1F). PD-L1 immunostaining can also help identify neoplastic cells in small biopsy samples [39]. Its expression on HRS cells indicates the key role of immune evasion in the tumorigenesis. More importantly, blocking the PD-1/PD-L1 axis is an efficient therapeutic approach for CHL [40,41], and PD-L1 expression level is associated with a good response to PD-1/PD-L1 inhibitors [42]. The prevalence of PD-L1 expression varies depending on the histologic subtype. Almost all NSCHL cases show PD-L1 expression on neoplastic cells, whereas other histological subtypes, especially LRCHL, express PD-L1 much less frequently [43,44]. 

HRS cells typically lack a B-cell phenotype, other than PAX5 expression, and usually are negative or show very weak staining for CD20, CD79a, OCT-2, and BOB.1 [45,46]. Nuclear PAX5 staining is usually weaker in HRS cells than in reactive B cells, which is a typical finding of CHL [46,47] (Figure 1G,H). However, improved antigen-retrieval techniques have led to increased frequency of detection of B-cell marker expression. In the current literature, CD20 expression has been observed in 20–50% of CHL [48,49,50]. In almost all cases, IRF4/MUM1 is expressed in RS cells, presumably due to NF-κB pathway activation; however, it has little significance in terms of diagnosis, since other lymphoid neoplasms mimicking CHLs also frequently express MUM1 [51]. BCL6 is expressed in around 20% of CHL cases [52]. 

### 2.5. Cellular Origin of Hodgkin Lymphoma 

For many years, the cellular origin of CHL has been controversial because the HRS cells of CHL have a morphology and immunophenotype that does not match any type of immune cells. Genetic analysis has revealed that in almost all CHL cases, the HRS cells have a clonally rearranged immunoglobulin (IG) gene [53]. Furthermore, a significant proportion of HRS cells have somatic hypermutation at the IG gene locus [53,54]. In around 30% of cases, IG gene rearrangements render the product nonfunctional through the introduction of stop codons, deletions generated within the GC [54]. In the other cases, HRS cells frequently lack Ig gene transcription ability due to functional defects in the Ig gene regulatory elements [53]. These genetic analysis findings indicate that HRS cells originate from GC cells, which normally cannot survive without B-cell receptor (BCR) signaling [55]. Notably, a minority of CHL cases have clonal TCR gene rearrangement and/or express T-cell markers [56]. Further research is needed to determine whether these minority cases represent T-cell-derived Hodgkin lymphoma, T-cell lymphomas mimicking CHL, or “B-cell” lymphomas with aberrant phenotypic/genetic changes.

### 2.6. Genetic Alterations

The most frequent genetic alteration found in CHL is a copy number gain of 9p24.1 at the locus including *PD-L1/L2* and *JAK2*, which is found in up to 97% of CHL cases [37]. A copy number gain of *PD-L1/L2* increases its transcripts in HRS cells [57]. Additionally, a copy number gain of *JAK2* leads to constitutive activation of JAK/STAT signaling, and thereby also induces PD-L1 expression on HRS cells [58]. A minority of CHL cases (4/200, 5%) exhibit unbalanced translocations involving 9p24.1, which might upregulate PD-L1 expression by stabilizing *PD-L1* mRNA [59]. An inactivating mutation of the Beta 2 microglobulin gene (*B2M*) is another prevalent gene mutation in CHL (up to 40%), which also contributes to escape from immune surveillance by CD8^+^ T cells, by limiting the cell surface expression of major histocompatibility complex class I (MHC class I) [60,61]. These immune-evasion-associated genetic alterations are more frequently observed in EBV^+^ CHL than in EBV^−^CHL, and presumably inhibit the T-cell response to the virus-derived antigen of EBV^+^CHL tumor cells [61].

In addition to *JAK2* copy number gain, the JAK-STAT pathway is also activated by inactivating mutations of *SOCS1* and *PTPN1*, which are both negative regulators of JAK/STAT signaling [61]. Activating mutation of *STAT6* is observed in~30% of CHL cases [62]. In total, almost all CHL cases harbor genetic alterations that affect the JAK/STAT pathway. 

CHL also frequently exhibits constitutive NF-κB signaling due to genetic alterations. A copy number gain of *REL*, a component of the NF-κB signaling pathway, is observed in over 50% of CHL cases, making this one of the most frequent copy number alterations in CHL [63]. Additionally, over 50% of CHL cases exhibit genetic deletion and/or inactivating mutations involving *TNFAIP3*, a negative regulator of the NF-κB pathway [64]. Truncations of other NF-κB regulator genes, including *NFKBIA* and *NFKBIE*, are less frequently reported [61]. 

Disruptive mutations of *GNA13* (encoding G protein subunit alpha-13) and ITPKB (encoding inositol-trisphosphate 3-kinase) are observed in around 30% of CHL [65], and reportedly induce Akt activation. Since Akt activation plays a central role in tonic BCR signaling, and rescues BCR knock-out in B-cell lymphoma cell lines [66], these genetic alterations may replace the function of BCR signaling among neoplastic cells in CHLs lacking BCR expression [67]. 

A recent study revealed that *ARID1A*, a member of the subunit of the chromatin remodeling SWItch/sucrose non-fermentable (SWI/SNF) complex, is truncated in 26% of CHL cases. It was suggested that *ARID1A* mutations may be a driver event of lymphomagenesis, and contribute to genomic instability of CHL [61]. 

Table 1 lists the representative genetic alterations of CHLs.

### 2.7. EBV Infection

It has been postulated that EBV plays an important role in the pathogenesis of CHLs. However, the prevalence of EBV positivity varies greatly depending on the histologic subtype. For example, EBV infection is detected in up to 75% and 65% of cases of MCCHL and LDCHL, respectively [76]. In contrast, NSCHL shows relatively low rates of EBV positivity (10–25%), and around half of LRCHL cases have EBV infection [77]. Intriguingly, NSCHL has a higher frequency of EBV positivity in elderly individuals (40%) compared to in young patients (10%), suggesting that NSCHL has different pathogenic mechanisms in elderly versus young patients [78]. EBV infection may substitute for the role of genetically altered intracellular pathways, as EBV^+^CHL cases show much lower numbers of somatic mutations compared with EBV^−^CHL [61,62]. For example, EBV^+^CHL cases express EBV-encoded latent membrane protein 1 (LMP1), which interacts with tumor necrosis factor receptor-associated factors and activates NF-κB signaling [79]. Consequently, EBV^+^CHL is less reliant on genetic aberrations that induce constitutively active NF-κB signaling [64]. Additionally, another EBV-coded latent membrane protein, LMP2a, mimics BCR signaling, and presumably rescues BCR-deficient B cells in the course of CHL development [80]. In normal B cells, EBV infection can induce the virus-replicative cycle and cell death. However, in HRS cells, virus-derived genes show a restricted pattern of expression, known as latency II, and entry to virus-replicative cycle is inhibited in HRS cells [81]. Latency II is characterized by the presence of Epstein–Barr virus nuclear antigen-1 (EBNA1) and LMP1 and LMP2 [82]. 

### 2.8. Microenvironment 

CHLs are characterized by the presence of an inflammatory background surrounding scattered neoplastic cells, and the relationship between the background immune cells and neoplastic cells is considered a key determinant of CHL disease characteristics. CHLs exploit immune evasion strategies to ensure their survival, indicating that specific features of the CHL microenvironment are associated with immune evasion mechanisms. CHLs contain heterogeneous inflammatory cell components, and recent advances in single-cell profiling methods have enabled the definition of each inflammatory cell subpopulation as a functional unit. 

Among the inflammatory cells in the background of CHL, CD4^+^ T cells are most enriched and their function has been well studied. CD4^+^ T cells in CHL exhibit Th1 polarization and increased terminal differentiation, and frequently express PD-1, indicating T-cell exhaustion [83]. They also express other exhaustion-associated proteins, such as TOX and TOX2 [84]. T-cell exhaustion can be induced by the PD-L1 molecule on tumor cells and by persistent tumor-specific antigen stimuli [85,86]. These T cells directly surround HRS cells, which is termed “rosetting”. The rosetting T cells provide a survival signal to HRS cells via surface expression of CD40L [83], and may also inhibit effector CD8^+^ T cells from direct contact with CHL cells [87]. Although the rosetting CD4^+^ T cells form an immunological synapse between CHL cells, and are likely activated by tumor-specific antigen, they do not expand due to immune regulatory mechanisms, such as immune checkpoint molecules and production of immunosuppressive cytokines [88]. The enrichment of type 1 regulatory (Tr1) T cells is another unique feature of CHL. Tr1 is a subset of LAG3-expressing regulatory T cells, which are induced in the periphery, not in the thymus [89]. CHL cell-derived cytokines reportedly induce Tr1 [89]. CD8^+^ T cells are outnumbered by CD4^+^ T cells in the tumor microenvironment (TME) of CHL, have follicular helper T-cell (Tfh)-like features, and are suggested to possess decreased cytotoxic function [90]. CD8^+^ T cells also express exhaustion-associated proteins, although this expression is weaker than in CD4^+^ T cells [89]. CD8^+^ T cells are functionally regulated by immunosuppressive molecules (such as TGF-β and Glectin-1 produced by HRS cells), overexpression of PD-L1 and PD-L2, and lack of MHC complex on HRS cells [91]. 

Tissue-associated macrophages also exert immune-regulatory functions in the TME. Tumor-associated macrophages (TAMs) express PD-L1 on the cell surface, which is an important source of PD-1/PD-L1 signaling in the TME [92]. Macrophages comprise two cell types: “M1-like” cells, which express CD68 and have anti-tumor effects, and “M2-like” cells, which express CD163 and have immunosuppressive functions [93]. TAMs in CHLs show skewed polarization toward the “M2-like” type, and this polarization is suggested to be induced by cytokines derived from CHL [94]. Non-malignant B cells comprise approximately half of the inflammatory cells in the TME; however, their biological contribution remains obscure. 

## 3. Nodular Lymphocyte-Predominant Hodgkin Lymphoma (NLPHL)

### 3.1. Epidemiology and Clinical Features

NLPHL comprises approximately 5–10% of CHL [95], has a peak incidence in the fourth decade of life, and exhibits a male predominance of 3:1. NLPHL most frequently presents as early-stage disease, without B symptoms [96]. It generally involves peripheral lymph nodes, such as the cervical, axillary, and inguinal lymph nodes, and rarely exhibits mediastinal involvement [97]. Bone marrow involvement has been reported in <10% of patients with NLPHL [98], and its presence is a significant inferior prognostic indicator [98]. In contrast to CHL, NLPHL tends to spare axial lymph nodes and preferentially involves peripheral lymph nodes. NLPHL has an excellent prognosis, with 10-year overall survival reaching around 90%, and radiotherapy without chemotherapy can be a therapeutic option in stage IA patients [99]. Sometimes NLPHL progresses into THRLBCL, and these two disease entities can even co-exist at a single site, suggesting that they are closely related [100,101].

### 3.2. Histology and Immunophenotype

**Figure 3:**
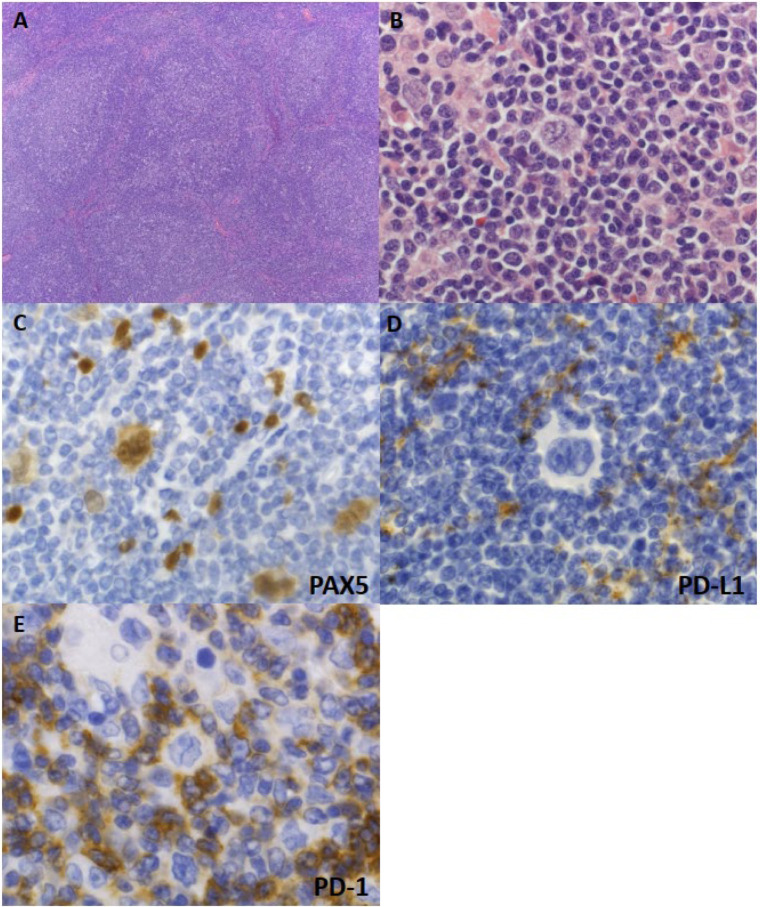
Histological and immunohistochemical features of nodular lymphocyte-predominant HL (NLPHL). (**A**) Vague nodular architecture is observed. (**B**) Scattered neoplastic cells feature polylobated (popcorn-like) nuclei, and are called lymphocyte-predominant (LP) cells. (**C**) LP cells show strong PAX5 expression. (**D**) LP cells lack PD-L1 expression (assessed using clone SP142). (**E**) LP cells are ringed by PD-1-positive “rosetting” T lymphocytes.

The neoplastic cells of NLPHL are termed lymphocyte-predominant (LP) cells, and are characterized by large polylobated nuclei with scant cytoplasm, referred to as “popcorn” cells. Some NLPHL cases involve LP cells with multinucleated nuclei and prominent nucleoli, which are indistinguishable from the HRS cells of CHLs [102]. The histological growth pattern of NLPHL can be divided into six subtypes, termed patterns A–F [103]. The most prevalent and prototypic pattern is pattern A—a “classical” B-cell-rich nodular pattern (Figure 3A,B), characterized by scattered LP cells ringed by CD57-positive T cells, against a nodular background comprising reactive small B cells. In pattern B, nodular architectures are interconnected, forming serpiginous shapes. In pattern C, LP cells extend outside of the nodules, against a background of reactive T cells. In pattern D, LP cells are scattered in the reactive nodules but, unlike in pattern A, these nodules are composed of small T cells. Pattern E is a diffuse (THRLBCL-like) pattern characterized by scattered LP cells, against a diffuse background of reactive T cells without CD57-positive T cells or a follicular dendritic cell (FDC) meshwork. Pattern F is a diffuse, moth-eaten, B-cell-rich pattern, which is reminiscent of the classic nodular pattern but lacks the formation of distinct nodules. Other than pattern E, all histological patterns have common nodular features with FDC meshwork [103]. FDC meshworks are filled with numerous small B cells (often with a small B-cell mantle), which can be used to differentiate from THRLBCL [104]. 

About half of NLPHL cases are composed of a single pure histological pattern, and the other half are composed of two or more histological patterns. Immunophenotypically, LP cells express pan-B-cell markers, including CD20, CD79a, PAX5, OCT2, and BOB.1 [105]. LP cells are typically positive for BCL6, a master regulator of GC formation, and negative for another GC marker, CD10 [106] (Figure 3C). In contrast to HRS cells, LP cells do not express CD30 or CD15, with rare exceptions [103,107,108]. Additionally, EBV infection is rarely observed in NLPHL. The latency of EBV in EBV^+^ NLPHL has not been determined, and LMP1 expression was frequently observed in EBV^+^ NLPHL [109]. The PD-L1 expression rate of LP cells significantly varies across studies, and may be affected by the specific anti-PD-L1 antibody clone used [36,43,110] (Figure 3D). In our preliminary data obtained using clone SP142, neoplastic PD-L1 positivity has not yet been detected among NLPHL cases. In patterns D and E, enrichment of CD163-positive “M-2 like” macrophages has been observed, and is suggested to be associated with disease progression [111].

### 3.3. Cellular Origin and Molecular Biology

Genetic analysis of isolated LP cells has revealed clonal IG gene rearrangement, and ongoing somatic hypermutation [112], indicating that LP cells are derived from GC B cells. Gene expression profiling of isolated LP cells also shows close similarities between LP cells and GC B cells [113]. Unlike CHLs, LP cells often exhibit functional IG gene rearrangement, and have retained their B-cell program [113]. About half of NLPHL cases have BCL6 translocation, which is not observed in CHLs [74]. Next-generation sequencing reveals that LP cells harbor silencing mutations in *SOCS1* in around 50% of cases, which leads to JAK-STAT pathway activation. *SKG1*, *DUSP2*, and *JUNB*, which are regarded as tumor-suppressor genes, are also reported to be mutated in around 50% of cases [72] (Table 1). Notably, these genetic alterations are also observed in THRLBCL [114]. Additionally, the gene expression signature of THRLBCL cells closely resembles that of LP cells, suggesting that these two disease entities may share a common pathogenesis [113]. An array comparative genomic hybridization study, employing laser-capture microdissection, further revealed that these diseases have common genetic events, such as 2p16.1 amplification, and loss of 2p11.2 and 9p11.2 [115]. *PD-L1* amplification, which is frequently observed in CHLs, has not been found in NLPHL [115,116]. Aoki et al. recently reported that PD-L1 expression and genetic aberration were more frequent in LRCHL than in other CHLs, and suggested that TGF-β production of HRS cells, and the corresponding enrichment of PD-1^+^CXCL13^+^ T cells, may shape the immune microenvironment of LRCHL [44]. Given the histological resemblance between NLPHL and LRCHL, such a mechanism may also contribute to the lymphomagenesis of NLPHL. Around 30% of NLPHL express IgD and these cases represent a distinct clinical subtype showing strong male predominance, younger age, cervical lymph node involvement, and superior prognosis [117,118]. Recently, Lorenz et al. demonstrated that IgD^+^ LP cells exhibit specific BCR binding to *Moraxella catarrhalis* antigen, suggesting that this infection may promote lymphomagenesis of IgD^+^ NLPHL [119]. 

## 4. Differential Diagnosis of Hodgkin Lymphomas

**Figure 4:**
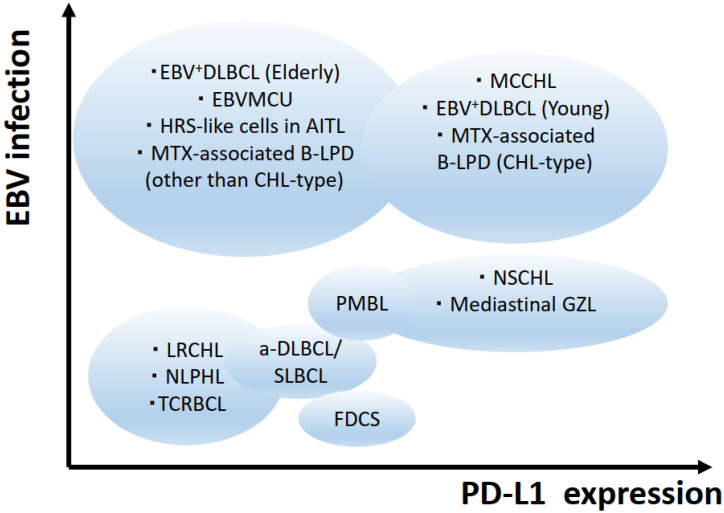
Images of the disease entities of classic Hodgkin lymphoma subtypes and its mimics. EBV = Epstein-Barr virus; AITL = angioimmunoblastic T-cell lymphoma; NSCHL = nodular sclerosis classic Hodgkin lymphoma; LRCHL = lymphocyte-rich classic Hodgkin lymphoma; MCCHL = mixed cellularity classic Hodgkin lymphoma; NLPHL = nodular lymphocyte-predominant Hodgkin lymphoma; DLBCL = diffuse large B-cell lymphoma; EBVMCU = EBV-positive mucocutaneous ulcer; MTX = methotrexate; LPD = lymphoproliferative disorder; PMBL = primary mediastinal large B-cell lymphoma; GZL = B-cell lymphoma unclassifiable, with features intermediate between DLBCL and CHL/Gray zone lymphoma; TCRBCL = T-cell/histiocyte-rich large B-cell lymphoma; a-DLBCL = anaplastic variant of DLBCL; SLBCL = sinusoidal large B-cell lymphoma; FDCS = follicular dendritic cell sarcoma.

HL diagnosis in routine pathological examination is often difficult, due to the histological diversity and overlapping features of HLs and HL mimics. Notably, common pathogenetic mechanisms are shared by HL and its mimics, as represented by NSCHL and mediastinal gray zone lymphoma. Recent studies have revealed that PD-L1 upregulation on neoplastic cells plays a central role in CHLs, especially NSCHL, and in other lymphoid neoplasms. EBV infection also plays a central role in the pathogenesis of CHLs, including MCCHL. Both PD-L1 immunohistochemistry (IHC) and EBV detection can be applied in routine pathological examinations, and are strongly associated with disease phenotype. Therefore, the diagnostic approach to HL and its mimics can be organized according to PD-L1 expression and EBV infection (Figure 4).

### 4.1. Differential Diagnosis of CHLs

#### 4.1.1. Primary Mediastinal Large B-Cell Lymphoma and B-Cell Lymphoma Unclassifiable, with Features Intermediate between DLBCL and CHL (Gray Zone Lymphoma)

Primary mediastinal large B-cell lymphoma (PMBL) and NSCHL share a number of common features, including preferential mediastinal involvement, female predominance, common occurrence in young adults, and large tumor cells with sclerosis. Sequential development of CHL and PMBL in the same patient has been reported, suggesting common pathogenesis of CHL and PMBL [120]. Gene expression profiling and genetic profiles further support a close relation between these two diseases [121]. Nevertheless, the therapeutic approach differs between these diseases, and thus CHL must be distinguished from PMBL for clinical management [122]. Unlike CHLs, the prototypical PMBL shows a monomorphous infiltrate of large cells with a sparse inflammatory background, and diffuse positive staining of pan-B-cell markers [123]. 

The differential diagnosis of CHL versus B-cell lymphoma unclassifiable, with features intermediate between DLBCL and CHL (mediastinal gray zone lymphoma: MGZL), is even more complicated. MGZL was originally proposed to be a disease representing the missing link between CHL and MLBCL [120]. Some MGZL cases exhibit a characteristic CHL immunophenotype with histologic appearance suggestive of MLBCL, while others show histology reminiscent of CHL with an immunophenotype suggestive of DLBCL, although the latter type lacks typical architectural features of CHL, including a nodular growth pattern and well-formed fibrous bands [124]. MGZL diagnosis is challenging for a pathologist, but the distinction of MGZL and CHL or PMBL is critical for clinical management. MGZL has a poorer prognosis than CHL, PMBL, and DLBCL, and it has been suggested that a DLBCL-based therapeutic regimen may be effective for MGZL. 

Our group has demonstrated that PD-L1 expression was more frequently observed in CHL than PMBL (100% vs. 18%), when using clone SP142 [125]. Seven NSCHL cases were positive for PD-L1, while only two out of 11 PMBL were positive for PD-L1. Notably, these PD-L1-positive PMBL cases were accompanied by a relatively rich inflammatory background, with a moderate number of small lymphocytes, and/or sclerotic changes, with difficulties to differentiate from MGZL [125]. Using the same antibody clone, a study by the Lymphoma Study Association (LYSA) also showed that neoplastic PD-L1 expression was more frequently observed in CHL than in MGZL (80% vs. 54%) [126]. Interestingly, our group recently reported composite lymphomas, comprising CHL with PD-L1 expression, and PMBL lacking PD-L1 [125]. The available data indicate that PD-L1 on neoplastic cells is involved in shaping the specific morphology and immune microenvironment of CHL. These cases also indicate that the differential diagnosis between CHL and MGZL or PMBL may sometimes be indistinguishable, especially from small biopsy samples. 

Non-mediastinal (systemic) GZL poses a more challenging diagnostic problem, and is characterized by onset in elderly patients, and more advanced disease than MGZL, without a bulky mass [126]. Recent studies reveal similar gene expression patterns between MGZL and CHL, as well as between non-mediastinal GZL and DLBCL [127,128]. Notably, non-mediastinal GZLs are characterized by a relatively low frequency of PD-L1 aberrations (up to 50%) [129], and by enrichment of *TP53* and *BCL2* mutations, and translocations of *BCL2* and/or *BCL6*, which are frequently detected in high-grade B-cell lymphomas transformed from low-grade B-cell lymphomas [130,131]. These findings suggest that non-mediastinal GZL includes transformed diseases from undiagnosed indolent malignancies, which have distinct pathogenesis from CHL, MGZL, and PMBL.

#### 4.1.2. DLBCL and Other B-Cell LPD with “HRS-like” Cells

The most problematic differential diagnosis of MCCHL, which is characterized by frequent EBV infection, is EBV-positive diffuse large B-cell lymphoma, not otherwise specified (EBV^+^DLBCL, NOS). This disease was formerly designated as EBV-positive DLBCL of the elderly in the WHO 2008 classification system. Later reports of EBV-positive large B-cell lymphoma in young patients [132] led to removal of the age denominator in the terminology of the 2017 WHO classification. However, the disease characteristics seem to differ between young and elderly patients. Young patients exhibit nodal lesions, and most show a THRLBCL-like histology, an excellent prognosis, and PD-L1 expression on neoplastic cells [132]. On the other hand, EBV-positive DLBCL in the elderly exhibits frequent extranodal manifestations, poor prognosis, and a low frequency of PD-L1 expression on neoplastic cells [133,134,135,136,137]. The neoplastic cells of EBV-positive DLBCL have a preserved B-cell program, and often exhibit an activated B-cell phenotype with expression of IRF4/MUM1 and CD30 [102,138].

EBV-positive mucocutaneous ulcer (EBVMCU) is a newly recognized EBV-associated B-cell lymphoproliferative disorder (LPD), which presents as sharply circumscribed isolated lesions that are confined to the oral mucosa, skin, and gastrointestinal tract, and which usually does not involve lymph nodes [139]. The lesions are often accompanied by EBV-infected B cells with HRS-like morphology. EBVMCU occurs in individuals with immunodeficient status (older age, immunosuppressant drug treatment, treated lymphoma, HIV infection, and primary immunodeficiencies), and is considered a specific type of immunodeficiency-associated LPD [140]. Unlike HRS cells in CHLs, the HRS-like cells in EBVMCU do not exhibit cell surface expression of PD-L1, with rare exceptions [140,141]. 

In patients with autoimmune disease treated by immunosuppressive agents, EBV-driven B-cell proliferation may occur with varying histologies (including DLBCL, polymorphic B-LPD, and CHL), and is called iatrogenic immunodeficiency-associated LPD. CHL-type iatrogenic immunodeficiency-associated LPD is histologically indistinguishable from CHLs in immunocompetent patients. Kohno et al. reported that PD-L1 expression on neoplastic cells was exclusively found in CHL-type methotrexate-associated LPD involving lymph nodes, while PD-L1 expression was not observed in other type of LPD [142]. 

These results suggest that immunosenescence/immunodeficiencies likely play a complementary role in the immune evasive mechanism of neoplastic cells in lymphomagenesis, given the low frequency of PD-L1 expression on the neoplastic cells in immunosenescent/immunodeficient patients. Indeed, the combination of neoplastic PD-L1 expression with immunosenescence/immunodeficiencies in a host appears to predict shorter progression-free survival, which is exemplified by PD-L1-expressing MTX-associated CHL-type LPD [136,142]. 

Aside from the abovementioned EBV-associated LPD or lymphoma, EBV-negative B-cell lymphoma may also contain HRS-like anaplastic cells. The anaplastic variant of DLBCL (A-DLBCL) is characterized by large, pleomorphic, and bizarre cells that often resemble Hodgkin/Reed–Sternberg (HRS) cells and hallmark cells of anaplastic large cell lymphoma. A-DLBCL overlaps with sinusoidal large B-cell lymphoma (SLBCL), in which CD30^+^ neoplastic cells show intrasinusoidal growth [143]. Junpeng et al. recently reported that SLBCL frequently harbors *PD-L1* amplifications and *TP53* aberrations, showing similarities to the genetic aberrations of non-mediastinal GZL, while gene mutations enriched in DLBCL are also found in A-DLBCL and SLBCL [144,145]. The underlying mechanisms of the histological similarities and PD-L1 expression of these disease are not yet well understood, and further studies are required.

#### 4.1.3. Anaplastic Large Cell Lymphoma, ALK-Positive and -Negative

Anaplastic large cell lymphoma (ALCL) is a mature T-cell neoplasm consisting of neoplastic cells with abundant cytoplasm and pleomorphic nuclei, and the diagnostic feature of horseshoe-shaped nuclei (“hallmark cell”). Around 80% of ALCL have chromosomal translocation t (2;5) involving *ALK* and *NPM,* and, less frequently, *DUSP22* and *TP63* translocations are also observed. ALCL is characterized by strong CD30 expression, and often lacks surface CD3 expression, while expressing one or more T-cell antigens, including CD2, CD4, and CD5. Neoplastic PD-L1 expression is consistently detected in ALK^+^ ALCL, while it is observed on the tumor cells of around 60% of ALK-negative ALCL cases. Their expression of PD-L1 is presumably induced by STAT3 activation [146]. ALCL frequently express epithelial membrane antigen (EMA) and CD43 (MT1) [147,148]. ALCL also frequently (around 80%) expresses cytotoxic molecules, such as TIA-1, Granzyme B, and/or perforin [149,150], posing a differential diagnostic problem vis à vis cytotoxic molecule-positive CHL, as reported by Asano et al. [56]. Cytotoxic molecule-positive CHL does not express B-cell antigens (including PAX5) but frequently expresses fascin, which may help in making an accurate diagnosis. The cell of origin of cytotoxic molecule-positive CHL remains unclear, as it lacks both T-cell-receptor gene rearrangement and IG gene rearrangement [56]. 

#### 4.1.4. T-Cell Lymphomas with “HRS-like” Cells

HRS-like cells are occasionally detected in several types of T-cell lymphoma, including angioimmunoblastic T-cell lymphoma (AITL) and nodal peripheral T-cell lymphoma of follicular helper T-cell type (PTCL-TFH), adult T-cell leukemia/lymphoma (ATLL), and chronic active EBV infection of the T-cell/natural killer (NK) cell type, systemic form (CAEBV-T/NK-S) [17]. In AITL, EBV-infected B cells are frequently observed in the background, and are thought to drive T-cell proliferation in the course of lymphomagenesis [151]. When neoplastic cells show minimal cellular atypia, T-cell lymphoma is difficult to diagnose and may be misdiagnosed as CHL. Sakakibara et al. recently reported that PD-L1 was rarely expressed in HRS-like cells in PTCL-TFH, which can help in making accurate diagnosis [43]. In ATLL, a Hodgkin-like variant accompanied by HRS-like cells has been reported, and these HRS-like cells are regarded as EBV-infected polyclonal B cells and non-neoplastic [152]. However, Karube et al. recently reported the identification of human T-cell leukemia virus type 1 (HTLV-1) RNA in HRS-like cells, and that HRS-like cells were true neoplastic cells in a significant number of cases [101]. Although the clinical significance of HRS-like cells in ATLL has not been determined, the HRS variant of ATLL should be kept in mind, especially in HTLV-1 endemic areas. 

#### 4.1.5. Dendritic Cell Neoplasms

Follicular dendritic cell sarcoma (FDCS) is a neoplastic proliferation of spindled-to-ovoid cells. Based on immunohistochemical findings, they are regarded as non-hematopoietic mesenchymal tumors, although they frequently involve lymph nodes [4]. It was recently discovered that a significant proportion of FDC sarcomas express PD-L1 on the neoplastic cells [153]. Some FDC sarcomas also exhibit clonal B-cell receptor rearrangement, genetic mutations inducing NF-κB activation, and/or JAK-STAT activation [154]. More intriguingly, some CHL cases reportedly express FDC markers [155]. These findings indicate biological relationships between CHL and FDCS. 

Interdigitating dendritic cell sarcoma (IDCS) is an extremely rare neoplasm that features pleomorphic large cells with indented nuclei and abundant cytoplasm [156]. Based on the ultrastructural findings, their postulated normal counterpart is interdigitating cells in lymph nodes. We recently reported a case of IDCS expressing PD-L1, which posed a challenging diagnostic problem vis à vis CHL [157]. Since neoplastic B cells are reportedly capable of differentiating into mesenchymal cells, it is possible that these PD-L1-expressing dendritic cell neoplasms are derived from malignant B cells that have experienced transdifferentiation, and that share pathogenetic mechanisms with CHLs. Notably, it has been reported that PD-L1-expressing FDCS exhibits a good response to immune checkpoint therapy, suggesting that dendritic cell neoplasms should be managed according to PD-L1 status for optimal clinical benefit [158]. Further studies are needed to clarify these issues.

### 4.2. Differential Diagnosis of NLPHL

Morphologically, NLPHL is highly similar to THRLBCL. In contrast to NLPHL, TCRBCL has an aggressive clinical course, and requires chemotherapy even in early stages. However, the distinction of a diffuse type of NLPHL from THRLBCL is difficult or may not be possible. In NLPHL showing a nodular pattern, the presence of FDC meshwork and CD57-positive or PD-1-positive T-cell rosettes can help in differentiation from THRLBCL [104,159] (Figure 3E). In the case of pure diffuse type lacking a nodular component, the lesion would be regarded as THRLBCL [103]. In rare cases, NLPHL contain background atypical T cells, and can histologically mimic a peripheral T-cell lymphoma. Such presentations are typically observed in young patients, such that the combination of young age, absence of pan-T-cell marker loss, and T-cell clonality might help with diagnosis as NLPHL [160].

## 5. Future Perspectives

Recent studies have revealed that HLs originate from GC B cells, and that immune evasive mechanisms play a central role in lymphomagenesis. PD-L1 overexpression on HRS cells is currently asserted to be a defining feature of HLs that preferentially affect immunological sites (LN, thymus, and spleen). In contrast to CHLs, PD-L1^+^ extranodal DLBCLs do exist. These entities involve extranodal sites, and include intravascular large B-cell lymphoma (IVL) and DLBCL involving immune sanctuary sites, such as the central nervous system, testes, and adrenal glands [136,161,162]. It has been suggested that these neoplasms acquire PD-L1 genetic aberrations in the GC process [163,164]. However, paradoxically, tumor cells with PD-L1 expression preferentially propagate in immune-sanctuary sites where tumor cells have little chance to encounter tumor-specific T-cells. The underlying mechanisms discriminating these lymphoid neoplasms and HLs have not yet been discovered. Notably, both CHL and non-Hodgkin lymphoma exhibit heterogeneous intratumoral or non-neoplastic expression of PD-L1. In such cases, there may be site-specific interaction between PD-L1-positive neoplastic cells and the environment, such as binding of PD-L1 and CD80 on endothelium [165].

Previous studies show that the presence of HRS cells is seemingly associated with PD-L1 expression, especially in EBV^−^CHLs. In the syncytial variant, PD-L1 expression is primarily found in HRS cells, but also rarely in small cohesive tumor cells [166]. It is currently unclear whether this heterogeneity is derived from genetic heterogeneity or transient expression induced by intracellular signaling, such as JAK/STAT activation. Moreover, while it is postulated that HRS cells represent genomic instability, the mechanisms of HRS cell formation and their association with PD-L1 expression remain largely unknown [167]. 

## 6. Conclusions

Genetic analysis of HLs has been difficult due to the rarity of HRS cells in the tumor tissue, and the lack of in vitro or in vivo models simulating the microenvironment of HLs. Newly developed technologies, such as single-cell sequencing, have enabled us to comprehensively analyze neoplastic cells and their microenvironment, leading to rapid expansion of our knowledge of HL biology. In this modern era of immune-oncology, CHLs may be hypothetically assumed to be lymphoid organ (LN, thymus, and spleen)-localized, immune escape-associated, lymphoid cell-driven neoplasms (mostly of B-cell origin). Nevertheless, there may be issues that remain to be addressed about the pathogenesis and classification of HLs. In particular, future studies are needed to guide further improvement of patient stratification strategies.

## Figures and Tables

**Table 1:** Representative genetic alterations of classic Hodgkin lymphomas and nodular lymphocyte-predominant Hodgkin lymphoma [37,61,62,64,68,69,70,71,72,73,74,75].

Function	Type of Genetic Alteration	Frequency (%)		Reference
		CHLs	NLPHL	
**Immune evasion**				
*PD-L1/PD-L1*	Gain/amplification	30–97		[37,44,61]
*B2M*	SNV, indel	39		[61]
*CIITA*	Translocation, SNV	8		[61]
**JAK/STAT activation**				
*JAK2*	Gain/amplification	33		[75]
*SOCS1*	SNV	40–70	50	[68]
*STAT6*	SNV, gain	30		[61,62]
*PTPN1*	SNV, indel	22		[61]
*XPO1*	SNV, gain	18–26		[62]
**Constitutive NF-κB activation**				
*TNFAIP3*	SNV, indel	57–74		[61,64]
*REL*	Gain/amplification	50	50	[63]
*NFKBIA*	SNV, indel	17		[61]
*NFKBIE*	SNV, indel	26		[61]
*NIK*	Gain/amplification	25		[67,70]
*BCL3*	Gain/translocation	15		[71]
**PI3K/AKT pathway activation**				
*GNA13*	SNV	24–26		[61,62]
*ITPKB*	SNV	16		[61,62]
**MAPK/ERK pathway activation**				
*DUSP2*	SNV		54	[72]
**AP-1 regulation**				
*JUNB*	SNV		39	[72]
**BCL6 disregulation**				
*BCL6*	Translocation		48	[73,74]
**Chromatin remodeling**				
*ARID1A*	SNV, indel	26		[61]
**Unknown function**				
*SGK1*	SNV		70	[72]

SNV: single nucleotide variant; CHL: classic Hodgkin lymphoma; NLPHL: nodular lymphocyte-predominant Hodgkin lymphoma.

## Data Availability

The data presented in this study are available on request from the corresponding author.
